# Tissue and serum lipidome shows altered lipid composition with diagnostic potential in mycosis fungoides

**DOI:** 10.18632/oncotarget.18228

**Published:** 2017-05-26

**Authors:** Chenchen Xu, Dan Zhou, Yixin Luo, Shuai Guo, Tao Wang, Jie Liu, Yuehua Liu, Zhili Li

**Affiliations:** ^1^ Department of Dermatology, Peking Union Medical College Hospital, Chinese Academy of Medical Sciences and Peking Union Medical College, Beijing 100730, China; ^2^ Department of Biophysics and Structural Biology, Institute of Basic Medical Sciences, Chinese Academy of Medical Sciences and Peking Union Medical College, Beijing 100005, China

**Keywords:** cutaneous T cell lymphoma, mycosis fungoides, mass spectrometry imaging, matrix-assisted laser desorption/ionization-Fourier transform ion cyclotron resonance mass spectrometry, lipidomics

## Abstract

Mycosis fungoides (MF) is the most common type of cutaneous T cell lymphoma. In this study, we used matrix-assisted laser desorption/ionization-Fourier transform ion cyclotron resonance mass spectrometry (MALDI-FTICR–MS) to perform lipidomic profiling of 5 MF tissue samples and 44 serum samples (22 from MF patients and 22 from control subjects). Multivariate statistical analysis of the mass spectral data showed that MF tissues had altered levels of seven lipids and MF sera had altered levels of twelve. Among these, six phosphotidylcholines, PC (34:2), PC (34:1), PC (36:3), PC (36:2), PC (32:0), and PC (38:4) and one sphingomyelin, SM (16:0) were altered in both MF tissues and sera. PC (34:2), PC (34:1), PC (36:3), and PC (36:2) levels were increased in both tissues and sera from MF patients, whereas SM (16:0), PC (32:0), and PC (38:4) levels were increased in MF sera but were decreased in MF tissues. We have thus identified multiple lipids that are altered in MF tissues and sera. This suggests serological and tissue lipidomic profiling could be an effective approach to screening for diagnostic biomarkers of MF.

## INTRODUCTION

Mycosis fungoides (MF) is the most common type of cutaneous T cell lymphoma (CTCL) [1]. It is often misdiagnosed as inflammatory skin disease because of scaly erythematous patches and plaques that are presented for years. However, the advanced form of MF is presented as generalized tumors involving the lymph nodes and inner viscera [2]. There is no effective treatment for advanced stage MF and the median survival time is less than 1.5 years. Therefore, new MF diagnostic biomarkers are necessary for early detection and clinical intervention to improve outcomes [3]. But, the etiology of MF is poorly understood. Aberrant expression and function of the many transcriptional factors and regulators of signal transduction has been reported in CTCL [4]. In most cases, the oncogenes activate downstream targets that are either directly or indirectly connected to the metabolism [5].

Lipidomics is an important branch of metabolomics used to profile lipid content and composition [6]. Lipid metabolism is closely associated with cell growth, proliferation, differentiation, and motility [7]. Therefore, lipidomal profile changes are generally associated with cancer, metabolic diseases, neurological diseases and inflammation. It is plausible that MF also involves changes in lipid metabolism that can be identified by lipidomics. Therefore, we postulated that lipidomic profiling may help identify lipid diagnostic biomarkers of CTCL. We opted for matrix-assisted laser desorption/ionization-mass spectrometry imaging (MALDI-MSI) that simultaneously maps multiple lipids while preserving the morphological integrity of the analyzed tissue [8]. MALDI coupled with Fourier transform ion cyclotron resonance mass spectrometry (FTICR– MS) has high resolution and mass accuracy that can greatly improve its reliability and validity [9].

Therefore, the aim of this study was to elucidate the expression profile of lipids in MF by MALDI-FTICR–MS imaging and identify potential tissue and serum biomarkers in MF.

## RESULTS

### Mass spectral imaging of MF tissue samples

**Figure 1:**
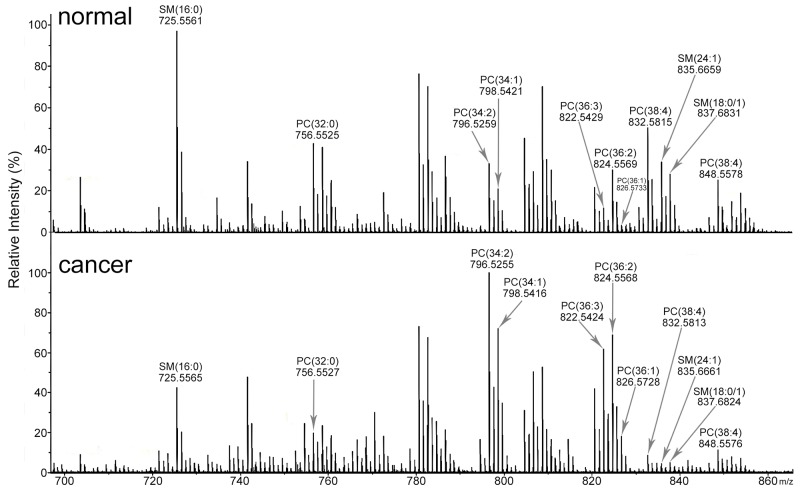
Mass spectra of lipids in MF tissue sections Representative mass spectra of MF tissue sections in positive ion mode showing differences in lipid composition between cancer (bottom) and adjacent non-cancer (top) areas.

**Figure 2:**
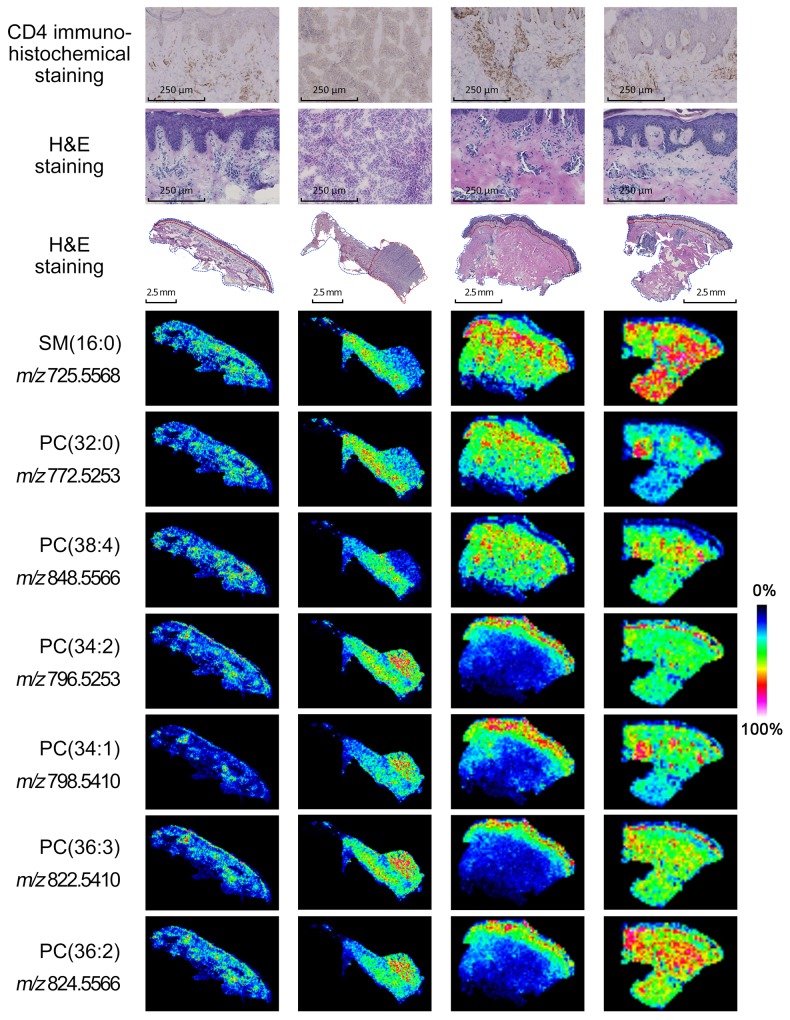
Mass spectrometry imaging of MF tissues Representative mass spectrometry images of 4 MF tissue samples (1, 3 4 and 5; left to right) showing ions SM (16:0) at m/z 725.5568, PC (32:0) at m/z 772.5253, PC (38:4) at m/z 848.5566, PC (34:2) at m/z 796.5253, PC (34:1) at m/z 798.5410, PC (36:3) at m/z 822.5410, and PC (36:2) at m/z 824.5566. The corresponding H&E and CD4 immunohistochemical staining is shown on top. The areas corresponding to cancer and adjacent non-cancer areas are shown by red and blue dotted lines, respectively. Note: The cell types in the non-cancer areas include keratinocytes, fibroblasts, fibers and matrix.

We used mass spectrometry imaging (MSI) in the positive ion mode to analyze the intensities of various phosphatidylcholines (PCs) and sphingomyelins (SMs) in the cancer and adjacent non-cancer areas in MF patient tissue sections. The ion intensities of PC (34:2) at *m/z* 796.5253, PC (34:1) at *m/z* 798.5410, PC (36:3) at *m/z* 822.5410, and PC (36:2) at *m/z* 824.5566 were higher in the cancer areas compared to the adjacent non-cancer areas (Figure 1). Also, the ion intensities of SM (16:0) at *m/z* 725.5568, PC (32:0) at *m/z* 772.5253, and PC (38:4) at *m/z* 848.5566 were lower in the tumor areas compared to the adjacent non-tumor areas (Figure 1). The images of H&E and CD4 immunohistochemical stained tissue sections are shown in Figure 2.

### Differentially expressed lipids in cancer areas of MF tissue samples

**Figure 3:**
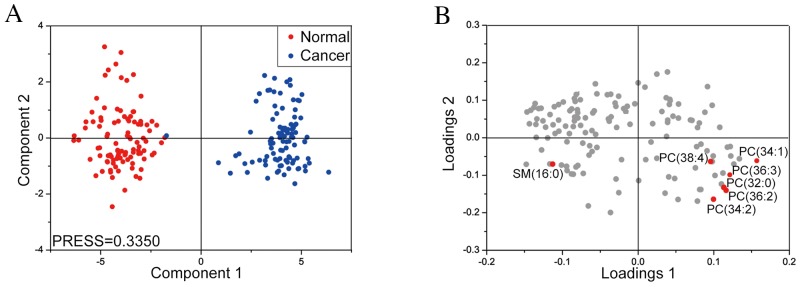
Partial least squares-discriminant analysis of lipid profiles in cancer and non-cancer areas of MF tissues (Left) Partial least squares-discriminant analysis (PLS-DA) score plot of component 2 versus component1 for cancer (blue dots) and adjacent non-cancer (red dots) areas from MF patient tissue samples. (Right) PLS-DA loading plot comparing variables (lipids) between cancer (loadings 1) and non-cancer areas (loadings 2) in MF patient tissue sections. The variables (lipids) in tissue samples with VIP values of more than 1.0 are marked in red.

To further confirm the lipid changes in MF, we collected twenty spectra (or pixels) at different positions of both MF cancer and non-cancer areas for each tissue sample. Then, we conducted multivariate statistical analysis of 138 lipids (or variables) to assess MF tissue heterogenity. The PLS-DA score plots showed differences between cancer (blue dots) and the adjacent non-cancer (red dots) areas with the predicted residual sum of square (PRESS) of 0.3350 (Figure 3A). These results demonstrated changes in lipid profile between the normal and MF cancer tissues. The differentially expressed lipids with VIP values of more than 1.0 were identified (Figure 3B). As shown in Table 1, the 7 differentially expressed lipids are SM (16:0), PC (32:0), PC (34:2), PC (34:1), PC (36:3), PC (36:2) and PC (38:4).

**Table 1:** Indentified important lipids from tissues and sera

Measured,*m/z*	Calculated,*m/z*	Error(ppm)	Ion form	Compound	Molecularformula	Structurally specific CID ions,*m/z*
482.3607	482.3605	0.39	[M+H]^+^	PC(O-16:0/0:0)	C24H52NO6P	
496.3401	496.3398	0.56	[M+H]^+^	LPC(16:0)	C24H50NO7P	
510.3556	510.3554	0.31	[M+H]^+^	LPE(20:0)	C25H52NO7P	
520.3399	520.3398	0.17	[M+H]^+^	LPC(18:2)	C26H50NO7P	
524.3711	524.3711	0.11	[M+H]^+^	LPC(18:0)	C26H54NO7P	
758.5691	758.5694	-0.41	[M+H]^+^	PC(34:2)	C42H80NO8P	
796.5252	796.5253	-0.18	[M+K]^+^	PC(34:2)	C42H80NO8P	613.4600/737.4512/796.5252
798.5409	798.5410	-0.14	[M+K]^+^	PC(34:1)	C42H82NO8P	615.4752/739.4690/798.5409
824.5564	824.5566	-0.22	[M+K]^+^	PC(36:2)	C44H84NO8P	641.4915/765.4838/824.5566
725.5566	725.5568	-0.32	[M+Na]^+^	SM(16:0)	C39H79N2O6P	542.4914/ 666.4845/725.5568
756.5509	756.5514	-0.58	[M+Na]^+^	PC(32:0)	C40H80NO8P	573.4856/697.4774/756.5514
772.5254	772.52532	0.16	[M+K]^+^	PC(32:0)	C40H80NO8P	
822.5408	822.5410	-0.21	[M+K]^+^	PC(36:3)	C44H82NO8P	639.4770/763.4685/822.5409
832.5820	832.5827	-0.86	[M+Na]^+^	PC(38:4)	C46H84NO8P	649.5164/773.5077/832.5826
848.5564	848.5566	-0.21	[M+K]^+^	PC(38:4)	C46H84NO8P	

### Differentially expressed lipids in MF patient sera

**Figure 4:**
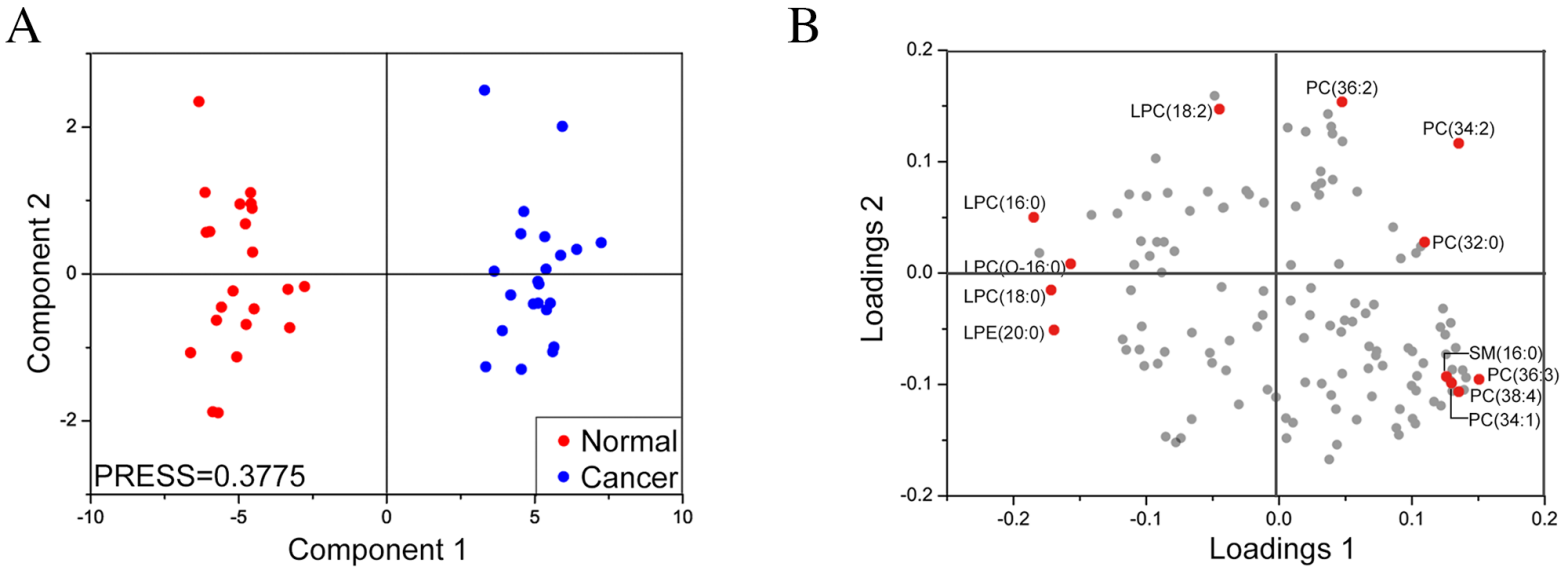
Partial least squares-discriminant analysis of lipid profiles of sera from MF and control subjects (Left) PLS-DA score plot of component 2 versus component 1 for serum samples from MF (blue dots) and control (red dots) subjects. (Right) PLS-DA loading plot comparing variables (lipids) between serum samples of MF and control subjects. The variables (lipids) in sera with VIP values of more than 1.0 are marked in red.

MALDI-FTICR MS analysis was conducted on serum samples from 22 MF and control subjects each. Then, multivariate analysis was performed for 130 lipids based on the mass spectrometry data. The PLS-DA score plots showed two clusters between MF (blue dots) and the control samples (red dots) with predicted residual sum of square (PRESS) score of 0.3775 (Figure 4A). This suggested changes in serum lipid profiles of MF patients. We identified 12 lipids with VIP values of more than 1.0 (Figure 4B). As shown in Table 1, these included lysophosphatidylcholines (LPCs) [LPC(16:0), LPC(18:0), LPC(18:2)], lysophosphatidylethanolamines (LPEs) [LPE(20:0)], PC(34:2), PC(O-16:0/0:0), PC(36:2), PC(32:0), PC(36:3), PC(38:4), PC(34:1) and SM(16:0).

### Identification of potential lipid diagnostic biomarkers in MF tissues and sera

**Figure 5:**
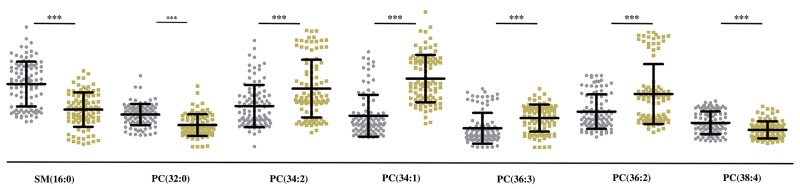
Quantitative analysis of 7 lipids in MF tissue samples Scatter plots show levels of 7 differentially expressed lipids in non-cancer (grey dots) and cancer (yellow dots) areas in MF patient tissues. *** denotes p<.001; ** denotes p<.01 and * denotes p<.05.

**Figure 6:**
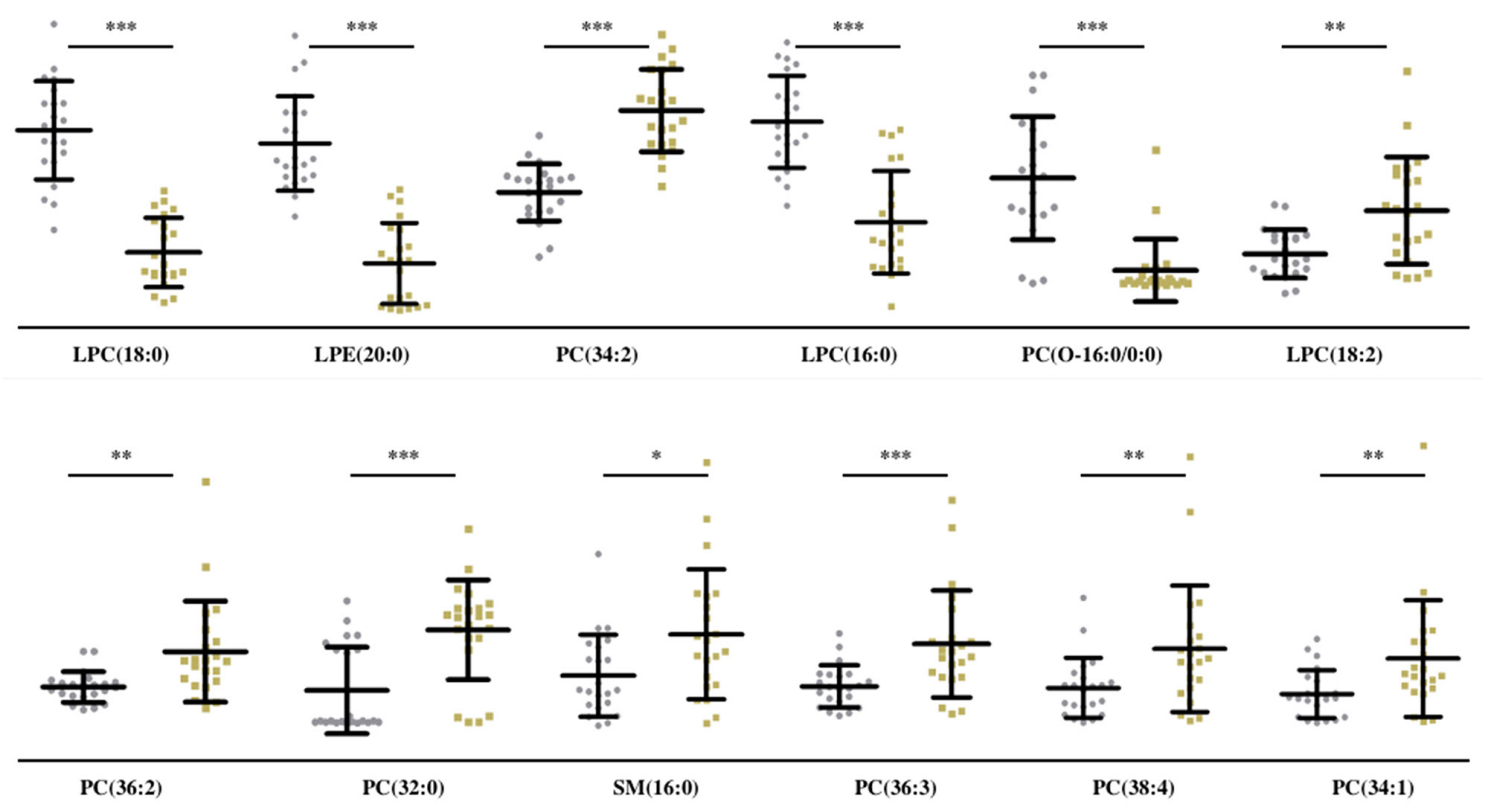
Quantitative analysis of 12 lipids in sera of MF and control subjects Scatter plots show levels of 12 differentially expressed lipids in sera from control (grey dots) and MF (yellow dots) subjects. *** denotes p<.001; ** denotes p<.01 and * denotes p<.05.

In tissues, we observed low levels of SM (16:0), PC (32:0), PC (38:4) and high levels of PC (34:2), PC (34:1), PC (36:3) and PC (36:2) in MF cancer areas compared to adjacent non-cancer areas (Figure 5). On the other hand, we observed low levels of PC (O-16:0/0:0), LPC (16:0), LPC (18:0) and LPE (20:0) and high levels of PC (34:2), LPC (18:2), PC (36:2), PC (32:0), SM (16:0), PC (36:3), PC (38:4), PC (34:1) in MF patients compared to control subjects (Figure 6).

We observed that PC (34:2), PC (34:1), PC (36:3), PC (36:2), SM (16:0), PC (32:0), and PC (38:4) changed in both tissues and sera of MF patients compared to control subjects. Interestingly, PC (34:2), PC (34:1), PC (36:3), and PC (36:2) increased in both tissues and sera of MF patients, whereas SM (16:0), PC (32:0), and PC (38:4) levels increased in the sera but decreased in the tissues of MF patients compared to control subjects.

## DISCUSSION

Mycosis fungoides accounts for 70-75% of all CTCLs. Patients with advanced MF have generalized tumors and a median survival of less than 1.5 years. Therefore, early diagnosis is of paramount importance. However, early diagnosis of MF is difficult due to non-specific clinical manifestation and lack of effective diagnostic biomarkers [3]. Therefore, we investigated lipid profile changes in the 5 MF tumor tissue samples and sera from 22 MF and 22 control subjects by highly reliable and sensitive MALDI-FTICR–MS technique. We identified 12 differentially expressed lipids in the MF tissues and 7 differentially expressed lipids in the MF patient sera. We also compared the differentially expressed lipids in the tissues and sera of MF patients and identified a subset of lipids that are potential diagnostic biomarkers.

Lipidomics was first conducted in 2003 to analyze lipid profiles in experimental samples [10]. There are nine categories of lipids including fatty acids, glycerolipids, sphingolipids, glycerophospholipids, sterol lipids, prenolipids, saccharolipids, polyketides and sterols. Lipids are involved in many physiological metabolic pathways such as cell fusion/division, apoptosis, shape, membrane vesicle trafficking and signal transduction [11]. Altered lipid content is associated with many human diseases including diabetes, neurodegenerative diseases, heart disease and cancer [11]. It is well established that tumor cells change metabolic profiles for growth, survival and differentiation [12]. Altered lipid profiles have been demonstrated in many cells and tissues [13, 14]. Some lipids have shown potential as tumor biomarkers. For example, increased polyunsaturated fatty acids (PUFAs) are observed in apoptosing glioma cells and in dedifferentiated and pleomorphic liposacomas [15, 16]. Furthermore, Zhou *et al* identified metabolic changes in the plasma of CTCL patients and mapped the potential biomarkers with ultrahigh performance liquid chromatography–quadrupole time-of-flight mass spectrometry (UHPLC-QTOF-MS) [17]. They observed that a few lipids like LysoPC (16:0), LPA (16:0/0:0) and LPA (0:0/16:0) were aberrantly expressed in CTCL plasma in positive and negative modes. However, systematic lipidomic study in MF had not been conducted previously. To our best knowledge, ours is the first study to analyze lipidomic changes in both MF tissue samples and sera.

Spectroscopic methods including mass spectrometry (MS) have been preferred to traditional histochemical stains in lipidomic investigations. This is because the common lipid stains are limited to few neutral lipids in tissues only [18]. Among the spectroscopy methods, MALDI–MSI is the preferred choice for *in situ* lipid research because it can be performed on a tissue section without destroying the tissue. MALDI–MSI can be used for molecular classification and histological diagnosis. In addition, it can predict prognosis, treatment response, or metastasis potential of cancers as well. MALDI–MSI has high spatial resolution and sensitivity, good salt tolerance and broad mass range. Since it can be performed without destroying the tissue sample, histological evaluation can be performed on the same tissue sample [19]. Tissue morphology is critical for MF tissue analysis because the malignant tumor cells co-exist with normal skin cells, immune cells, blood vessels, connective tissue and other micro-environmental factors. MALDI–MSI has been used to study brain, breast, lung, ovarian, prostate, and gastrointestinal cancers [20]. However, very few studies on MF tissue samples are based on MALDI–MSI. Hence, we used MALDI–MSI to investigate lipidomic changes in MF tissue and sera samples.

Another crucial aspects in our study was using adjacent non-cancer area as control. This reduced the potential confounding variations due to diet, skin type, age, sex, genetic background, metabolic status, and environmental influences. Serum lipid profiling was also performed by MALDI-FTICR–MS in our study. We predicted that lipids that changed significantly in both tumor tissues and sera were potential diagnostic biomarkers. We also compared the lipid profiles in MF tissues and sera to gain further insights regarding our findings.

Phosphatidylcholine (PC) is abundant in cell membranes and involved in key cellular signaling pathways [21]. Aberrant phospholipid metabolism is also established in many cancers. Increased phospholipid content is demonstrated in transformed cells and during tumor progression [21]. Increased PC was also shown in breast cancer tissue compared to adjacent normal breast tissue [22]. In our study, PC (34:2), PC (34:1), PC (36:3), and PC (36:2) increased significantly between MF and normal tissue and sera. However, PC (32:0), and PC (38:4) decreased in MF tissue compared to adjacent non-tumor tissue.

Sphingomyelins (SMs) are lipids that regulate many cellular functions, since they transmit the transmembrane signals through microdomains and the intracellular vesicular trafficking [23]. Also, SMs modulate cancer cell death. Studies have shown that some SMs promote tumorigenesis, whereas others repress it [24]. In this study, we observed low levels of SM (16:0) in MF tissues. However, in the sera, we observed high levels of SM (16:0) in MF patients. The reason for these differences is not known and need to be further investigated.

In summary, we comprehensively analyzed the lipid profiles of MF patient tissues and sera by MALDI-FTICR–MS. We found altered lipid composition in both tissue and sera of MF patients and identified few lipids with diagnostic potential. Our study also showed that serological lipidomic profiling combined with tissue MSI is a powerful tool for screening novel diagnostic biomarkers.

## MATERIALS AND METHODS

### Patient recruitment and clinical sample collection

This study was approved by the Ethics Committee of Institute of Peking Union Medical College Hospital, Chinese Academy of Medical Sciences. Informed consent was obtained from all participants. Tissue samples were obtained from 5 MF patients from Peking Union Medical College Hospital (Beijing, China) after clinical, pathological, immunohistochemical and TCR gene rearrangement investigations were completed and confirmed. The tissue samples were snap-frozen in liquid nitrogen after washing with normal saline, and stored at -80°C until further experimentation. Serum samples were collected from 22 MF patients enrolled at Peking Union Medical College Hospital (Beijing, China) and 22 age and sex matched healthy volunteers as controls after clinical laboratory tests or routine physical examinations. The serum samples were stored at 4°C for <12h and then transferred to a -80°C freezer until further use. All the tissue and serum samples were obtained before clinical treatment. The patients were staged according to TNMB staging system and were evaluated and diagnosed by two trained dermatologists. The detailed information is listed in Tables 2 and 3.

**Table 2:** Clinical information of tissue and serum samples of MF patients

Sample type	No.	Sex	Age	TNMB stage
Tissue	1	F	70	T3N0M0B0
	2	F	43	T3N1M0B0
	3	F	35	T2N0M0B0
	4	F	44	T2N0M0B0
	5	M	42	T2N1M0B0
Sera	1	F	33	T2N0M0B0
	2	M	22	T2N0M0B0
	3	M	25	T2N1M0B0
	4	F	55	T2N1M0B0
	5	F	25	T2N0M0B0
	6	M	28	T2N0M0B0
	7	M	46	T2N0M0B0
	8	F	67	T2N0M0B0
	9	M	49	T2N0M0B0
	10	F	42	T2N1M0B0
	11	F	70	T3N0M0B0
	12	M	19	T2N0M0B0
	13	F	27	T2N0M0B0
	14	M	8	T2N0M0B0
	15	F	43	T3N0M0B0
	16	F	20	T2N0M0B0
	17	M	25	T2N0M0B0
	18	M	14	T2N0M0B0
	19	F	34	T3N0M0B0
	20	F	43	T3N1M0B0
	21	M	27	T3N0M0B0
	22	F	30	T4N1M0B0

(Tissue No.1 and sera No.11 came from same patient. Tissue No.2 and sera No.20 came from same patient.)

**Table 3:** Clinical information of serum samples

	No.	Sex (female/male)	Age (mean±SD)
MF	22	12/10	34.18±16.12
Normal	22	12/10	35.64±15.44

### Patient tissue sample preparation for MALDI-FTICR–MS

Three 12μm thick tissue sections were cut from snap-frozen patient tissue samples. Of these, 2 were affixed onto glass slides and stained with either hematoxylin and eosin (H&E) or CD4 immunohistochemical staining to determine cancer and non-cancer areas in each MF patient samples. The third section was thawed and mounted on a glass slide coated with indium tin oxide (ITO). Then, the tissue sample was immersed in 200μL of 2,5-dihydroxybenzoic acid (2,5-DHB; Sigma-Aldrich) solution (10 mg/ml in methanol/ water (50/50, v/v)) as matrix for efficient ionization and enhance sensitivity of MALDI-FTICR–MS as previously described [25].

### Serum sample preparation for MALDI-FTICR–MS

The serum samples were processed by first precipitating the serum proteins by mixing 50μL of each serum sample with 950μL of methanol/acetonitrile (3/2 ratio, v/v). The mixture was vortexed for 1min and stored at 4°C overnight followed by centrifugation at 19000g for 30 min to collect the supernatant. Then, 250μL dichloromethane and 100μL ultrapure water (18.2 MΩ/cm) were added to 50μL of the supernatant in a glass vial and mixed by vortexing for 30s. The mixture was centrifuged at 1250g for 6min. Then, 75μL of the supernatant was transferred into a new glass vial and air-dried at room temperature. The dried samples were stored at −80°C until further use. For MALDI–MS analysis, the dried sample was redissolved by 150μL of 50% methanol in ultrapure water (v/v). Then, 0.2μL of the solution was spotted onto a MTP 384 polished steel plate (BrukerDaltonics, Billerica, MA, USA), air-dried at room temperature, and then layered with 0.2μL of 10mg/ml 2,5-DHB in methanol/water/formic acid (50/50/0.1, v/v/v) solution.

### Mass spectrometry

MSI or mass spectrometry profiling was performed in a 9.4 T Apex-ultra hybrid Qh-FTICR mass spectrometer (Bruker Daltonics, Billerica, MA, USA) equipped with a 355 nm Nd/YAG Smartbeam laser (200 Hz). Mass spectra were acquired over the *m/z* range of 600-1000 with a mass resolution of 400,000 at *m/z* 400 in positive ion mode. For tissue analysis, mass spectrum at each pixel was conducted with two full scans with 70 laser shots each. MSI experiments were performed with a spatial resolution of 200μm. For serum analysis, ten spectra were obtained for each sample with 50 laser shots for each scan.

### MS data processing and statistical analysis

MS images were visualized using the FlexImaging software (version 2.1, Bruker Daltonics) with relative ion intensities. Instrument calibration was performed against four references (*m/z* 725.5568, *m/z* 796.5253,*m/z* 824.5566, and *m/z* 832.5827). Mass spectral data for tissue imaging and serum lipid analysis was obtained from the Apex Control 3.0.0 software (Bruker Daltonics, Billerica, MA, USA). Peaks with signal-to-noise ratio of >3, relative intensity of >0.1%, and absolute intensity threshold of more than 10000 were selected as reliable variables with reliable isotope distribution of less than 2% relative standard deviation between the experimental and theoretical values using DataAnalysis 4.0 software (Bruker Daltonics). After isotopic deconvolution, these peaks were aligned within a mass tolerance window (±0.001 Da) as a single variable among different samples. The [M+H]^+^, [M+Na]^+^, and [M+K]^+^ions were also combined as one variable. Missing values for some variables were replaced with the half the baseline strength in each spectrum. The intensities of all variables from one mass spectrum were normalized to a constant number of 1000.

The resulting dataset was transferred to a Microsoft Excel file (Microsoft, Redmond, WA, USA) for statistical analysis. SAS software (version 9.2, SAS Institute Inc., USA) was used for partial least squares discriminant analysis (PLS-DA). The predicted residual sum of square (PRESS) of PLS-DA was used to evaluate the model. Variables with p value of less than 0.05 (Wilcoxon- Mann-Whitney test) and variable importance in projection (VIP) of more than 1.0 were considered statistically significant.

### Structure identification

The lipids of interest in tissues and sera were identified using HMDB (http://www.hmdb.ca), and LIPID MAPS (http://www.lipidmaps.org/tools/index.html) databases based on their molecular mass, isotope distribution, and collision-induced dissociation tandem mass spectral data. The mass error was <2 ppm and the relative intensity error of their isotopic peaks between experimental and theoretical values was <2%.

## Abbreviations

MF: mycosis fungoides
CTCL: cutaneous T cell lymphoma
MALDI: matrix-assisted laser desorption/ionization
FTICR: fourier transform ion cyclotron resonance
MSI: mass spectrometry imaging
MS: mass spectrometry
PC: phosphatidylcholine
SM: sphingomyelin
LPC: lysophosphatidylcholine
LPE: lysophosphatidylethanolamine
PUFA: polyunsaturated fatty acid
UHPLC-QTOF-MS: ultrahigh performance liquid chromatography–quadrupole time-of-flight mass spectrometry
H&E: hematoxylin and eosin
ITO: indium tin oxide
2,5-DHB: 2,5- dihydroxybenzoic acid
PLS-DA: partial least squares discriminant analysis
PRESS: predicted residual sum of square
VIP: variable importance in the projection
